# The critical interaction between valproate sodium and warfarin: case report and review

**DOI:** 10.1186/s40360-018-0251-0

**Published:** 2018-10-01

**Authors:** Chenguang Zhou, Yi Sui, Weijin Zhao, Chunyao Dong, Li Ren, Pingmei Song, Bing Xu, Xiaohong Sun

**Affiliations:** 1 Department of Neurology, The Fifth Affiliated Hospital of Zhengzhou University, Zhengzhou, China; 2 Department of Neurology, Shenyang First People’s Hospital, Shenyang Brain Institute, Shenyang Medical College Affiliated Shenyang Brain Hospital, Shenyang, China; 3 Department of Diagnostic Sonography, Shenyang First People’s Hospital, Shenyang Brain Institute, Shenyang Medical College Affiliated Shenyang Brain Hospital, Shenyang, China; 4 Department of Neurology, the Fourth Affiliated Hospital, China Medical University, Shenyang, China

**Keywords:** Valproic acid, Warfarin, Interaction, International normalized ratio, Epilepsy

## Abstract

**Background:**

Valproic acid (VPA) and warfarin are commonly prescribed for patients with epilepsy and concomitant atrial fibrillation (AF). When VPA and warfarin are prescribed together, clinically important interactions may occur. VPA may replace warfarin from the protein binding sites and result in an abnormally increased anticoagulation effect. This is commonly underrecognized.

**Case presentation:**

In our case, we report a 78-year-old woman with a glioma who presented with status epilepticus. The patient was on warfarin to prevent cardiogenic embolism secondary to AF. Intravenous loading dose of VPA was administered, but international normalized ratio (INR) increased significantly to 8.26. Intravenous vitamin K1 was then given and the patient developed no overt bleeding during the hospitalization.

**Conclusion:**

By reviewing the literature and discussing the critical interaction between valproate sodium and warfarin, we conclude that intravenous VPA and the co-administrated warfarin may develop critical but underrecognized complications due to effects on the function of hepatic enzymes and displacement of protein binding sites.

## Background

Comorbidity associated with polypharmacy is increasingly common among older patients with epilepsy [1]. Antiepileptic drugs (AEDs) and co-administration of other medications may have clinically important implications due to their effects on the function of hepatic enzymes and displacement of protein binding sites [2]. The potential for an interaction between valproic acid (VPA) and warfarin has rarely been reported or discussed and in this study, we present an interaction between intravenous VPA and oral warfarin that resulted in a substantial increase in the international normalized ratio (INR).

## Case presentation

A 78-year-old woman was admitted to the Department of Neurology on the third occurrence of generalized tonic clonic seizures (GTCS). A glioma had been diagnosed, and resection was performed 5 years previously. Following surgery, levetiracetam (LEV), 500 mg once daily was prescribed but discontinued by the patient 1 month later. The past medical history was otherwise unremarkable, except for 14 years of warfarin use at 1.875 mg per day prescribed for the secondary prevention of embolic events from paroxysmal atrial fibrillation (AF). The patient’s INR had not been monitored for 6 months, but there was no overt bleeding.

On the day of admission, 10 mg of diazepam was given intravenously to terminate a five-minute of GTCS while *en route* to a brain computerized tomography (CT) scan. The working diagnosis was status epilepticus (SE), and a loading dose of intravenous valproate sodium (1200 mg) was administrated to relieve the recurrent GTCS and frequent focal aware seizures. The patient remained physically well during interictal phase. Oral LEV of 500 mg twice daily was prescribed when the patient had regained consciousness. Oral warfarin was not discontinued based on the initial INR of 2.02. The patient was also on 40 mg oral isosorbide mononitrate sustained release tablets once daily and 12.5 mg succinate metoprolol tablets twice daily as needed. The brain CT scan showed left frontal and parietal craniectomy and encephalomalacia at the left frontal lobe.

On the second day of admission, routine laboratory studies revealed otherwise unremarkable results, including PT 22.70 s, PT% 36, albumin 38.5 g/L, total protein 59.50 g/L, TBIL 41.6 μmol/L, DBIL 7.10 μmol/L, IBIL 34.50 μmol/L, LDH 243 U/L, and NT-pro BNP 1906 pg/ml. ECG showed paroxysmal AF with a ventricular rate of 73 bpm. Carotid Doppler ultrasonography showed hypoechoic plaques on the anterior wall of the bifurcation of the right common carotid artery (Table 1).

**Table 1:** Abnormal laboratory results on Day 2

Items	Results or descriptions
Albumin	38.5 g/L
Total protein	59.50 g/L
TBIL	41.6 μmol/L
DBIL	7.10 μmol/L
IBIL	34.50 μmol/L
LDH	243 U/L
NT-pro BNP	1906 pg/ml
ECG	paroxysmal AF with a ventricular rate of 73 bpm
Carotid Doppler ultrasonography	hypoechoic plaques on the anterior wall of the bifurcation of the right common carotid artery

The intravenous valproate sodium was discontinued at 44 h after admission, and a total dosage of which was approximately 2200 mg. Although seizure ceased from the start of VPA infusion, 18 h of video-monitoring electroencephalogram (EEG) commenced 5 h later. This demonstrated global interictal θ waves (4-7 Hz), but without epileptiform discharges.

On the 3rd hospital day, the patient developed a renal dysfunction based on the results of BUN 13.74 mmol/l and serum creatinine 126 μmol/l, which was attributed to insufficient fluid intake. Coincidentally, the INR was found to be 8.26, and INR 9 h later was 5.52. Oral warfarin was paused but was mistakenly given again 6 h later by a caregiver. A third INR revealed an almost identical value of 5.32. Following consultation with a hematologist, 5 mg of vitamin K1 was given intravenously and the INR 8 h later was 2.16. The patient remained asymptomatic and without evident bleeding. A repeat brain CT scan revealed no intracranial hemorrhage. The renal dysfunction was corrected on the 4th day. The repeat BUN and serum creatinine were 9.33 mmol/l and 79 μmol/l, respectively. Based on the observation of an INR of 2.16, warfarin was carefully restarted with a dose of 1.25 mg and titrated back to 1.875 mg with steady monitoring of the INR to ensure a value between 2 and 3 (Table 2).

**Table 2:** Medications and changes of key laboratory abnormalities

	Premorbidity	Day 1	Day 2	Day 3	Day 4	Day 5	Discharge
Warfarin PO	1.875 mg QD	1.875 mg QD	1.875 mg QD	paused	1.25 mg QD	1.875 mg QD	1.875 mg QD
Diazepam IV		10 mg					
LEV PO	500 mg QD	500 mg BID	500 mg BID	500 mg BID	500 mg BID	500 mg BID	500 mg BID
Valproate sodium IV		1200 mg Discontinuation at accumulated dose of 2200 mg					
Vitamin K1 IV				5 mg			
Other medications	isosorbide mononitrate sustained release tablets 40 mg PO QD, succinate metoprolol tablets 12.5 mg PO BID						
INR (time)		2.02 (10:51)	2.04 (09:30)	8.26 (10:56) 5.52 (16:11) 5.32 (22:41)	2.16 (06:18)	2.07 (09:34)	2.43 (10:01)
PT (seconds)		22.50	22.70	86.90 63.00 60.80	24.20	23.30	27.50
Creatinine (μmol/l)		75		126	79	64	58
Albumin (g/L)			38.50				31.60

*LEV* levetiracetam, *IV* intravenous, *INR* international normalized ratio, *PT* prothrombin time, *QD* once daily, *BID* twice daily, *PO* by mouth

## Discussion and conclusion

Prior to this case, we had noted that warfarin could interact with many AEDs, such as carbamazepine, phenytoin, and others. The theoretical drug-drug interaction between warfarin and VPA has been hypothesized and previously proposed [3, 4], but the real-world interaction between these two drugs has been scarcely discussed. Previously, Yoon et al. reported a similar case of an unexpected and substantial increase of INR due to the co-administration of valproate and warfarin [5]. Interestingly, both Yoon et al. and our study share the similarity that both of the patients were coincidently given LEV, either orally or intravenously. The mechanism of action of LEV appears to involve neuronal binding to synaptic vesicle protein 2A, inhibiting calcium release from intraneuronal vesicles and N-type calcium channels [6]. The metabolism of LEV is mostly independent of the CYP enzyme system, and LEV is neither an inducer nor inhibitor of these enzymes [6, 7]. In addition, LEV at a dose of 2000 mg/d does not affect the pharmacokinetics of warfarin [8]. In short, an interaction between LEV and warfarin seems unlikely, although we cannot entirely exclude any synergistic effects of LEV and VPA on warfarin or the potentiation of LEV regarding the interaction between VPA and warfarin. Except for VPA and warfarin, the only medications administered to the patient during this admission were intravenous diazepam, oral levetiracetam, isosorbide mononitrate sustained release tablets and succinate metoprolol tablets. No clinical-relevant interactions between warfarin and these medications have been documented or identified in the literature or drug interaction database. Importantly, no other medications, in particular vasoactive drugs, proton pump inhibitors or antibiotics were administered immediately before or during hospitalization.

Warfarin is composed of two racemic active forms, R-enantiomers and S-enantiomers, serum depletion and metabolism of which largely depends on hepatic enzymes [9, 10]. R-isomers are 2–5 times less potent than their S-isomer counterparts [11]. More specifically, inactivation and metabolism of R-enantiomers occur via CYP1A2 and CYP3A4, while that of S-enantiomers occur via CYP2C9 [12]. Inducers or inhibitors of these enzymes may influence the metabolism of warfarin and, thereafter, its blood concentration. For instance, amiodarone is a CYP2C9 inhibitor that can enhance warfarin’s anticoagulation effect [13], and carbamazepine is an CYP2C9 inducer that reduces its anticoagulant effect [14]. Likewise, VPA is an inhibitor of CYP2C9, uridine diphosphate glucuronosyltransferase, and epoxide hydrolase [15]. Presumably, activity of the CYP2C9 enzyme is inhibited by VPA, and as a result, S-warfarin inactivation is lessened and its serum concentration is accordingly increased. However, why intravenous rather than oral administration of valproate has this effect remains elusive.

This influence of CYP inhibitors on the metabolism of warfarin usually initiates within 24 h after addition of the inhibitor, but the time to maximal affect depends on the time at which both interacting drugs reach their steady state [15]. Although warfarin has an average half-life of 40 h [9], based on our observation in this case, the interaction between valproate and warfarin may be transient, since the INR value decreased dramatically from 8.26 to 5.52 within 8 h without any intervention.

Given that intravenous VPA is more than 85% protein bound and oral warfarin is 95% protein bound at therapeutic concentrations [16, 17], displacement of warfarin from its protein binding sites and subsequent redistribution may also account for their interaction [15, 18, 19]. In this regard, Rolan PE et al. proposed that intravenous agents with high protein-binding rates, high hepatic extraction ratios, and narrow therapeutic windows may exhibit clinically significant interactions by way of competitive displacement [19].

Impaired renal function, in particular chronic kidney disease, is associated with increased bleeding risk in older adults using warfarin [20–22]. Although specific mechanisms are not well understood, animal studies suggest that downregulation of cytochrome enzymes may alter the pharmacokinetics of warfarin in the presence of renal dysfunction [23, 24]. Specifically for this patient, hypoalbuminemia may also increase levels of unbound warfarin, INR and bleeding risk [25, 26]. According to a cross-sectional analysis, Limdi NA and colleagues suggested a 9.5% and 19% lower dose of warfarin, respectively for patients with the estimated glomerular filtration rate (eGFR) of 30–59 mL/min/1.73 m^2^ and eGFR < 30 mL/min/1.73 m^2^ [27].

Therapeutic drug monitoring can be particularly helpful in AED-treated elderly patients with conditions such as renal/liver disease or low albumin concentrations, trading off between seizure control and adverse effects [28–32]. Specifically for this patient, detection of high serum concentrations of VPA may account for, at least partly, the abovementioned competitive displacement of protein-binding sites or inhibition of CYP2C9 enzyme with warfarin. However, because VPA is a highly and saturable protein binding drug with 30% of free fraction at a concentration of 150 mg/L [30, 33], measurement of protein unbound VPA instead may reflect the true levels of its free components. In fact, measurement of unbound concentrations for highly protein bound AEDs such as VPA and phenytoin (i.e., > 90%) has been increasingly recommended [30–32, 34]. Unfortunately, neither total nor unbound concentration of VPA was examined in this patient due to guardian’s disconsent.

VPA is effective for treating focal and generalized epilepsies with well-established tolerability. An injectable formulation has been available since 1993, and the most commonly effective doses for treating SE vary between 15 and 45 mg/kg in bolus (6 or 10 mg/kg/min) followed by 1–3 mg/kg/h infiltration [35–39]. The most reported adverse effects of intravenous VPA is dizziness, thrombocytopenia, and hypotension [38].

Comorbidity has been increasingly noted among older individuals newly diagnosed with epilepsy. Of 259 patients on oral VPA in a registry, 29% had AF, 36% were receiving warfarin for various reasons [1] and importantly, 19% of enzyme-inducing AED users reported concomitant usage of warfarin. Besides the INR enhancement we described in this case, VPA may also cause thrombocytopenia and further impair platelet aggregation [40]. Therefore, when physicians decide to start VPA, particularly intravenously, in patients who are simultaneously on warfarin, caution should be taken and the INR should be closely monitored. Optimally, from our point of view, concurrent use of warfarin and intravenous VPA should be avoided, either by replacing warfarin with new oral anticoagulants (NOACs) or substituting intravenous VPA with benzodiazepines, such as lorazepam or diazepam. The efficacy of intravenous diazepam for controlling generalized convulsive status epilepticus is not different than that of VPA, despite its potentiation in regard to respiratory depression or hypotension [35, 41].

In conclusion, we reported an AF patient on oral warfarin presenting with SE and the intravenous VPA was used to control the epileptic seizures. We observed a significant INR level elevation in this patient. We conclude that intravenous VPA and the co-administrated warfarin may have clinically important implications due to effects on the function of hepatic enzymes and displacement of protein binding sites.

## Abbreviations

AEDs: Antiepileptic drugs
AF: Atrial fibrillation
BID: Twice daily
BNP: Brain natriuretic peptide
CT: Computerized tomography
CYP: Cytochrome P450
DBIL: Direct bilirubin
EEG: Electroencephalogram
eGFR: Estimated glomerular filtration rate
GTCS: Generalized tonic clonic seizures
IBIL: Indirect bilirubin
INR: International normalized ratio
LDH: Lactate dehydrogenase
LEV: Levetiracetam
NOACs: New oral anticoagulants
PO: By mouth
PT: Prothrombin time
QD: Once daily
SE: Status epilepticus
TBIL: Total bilirubin
VPA: Valproic acid
